# Consulting with a folk deity before making decisions: spiritual practices in parents facing end-of-life decisions for their child on life support with brain stem dysfunction

**DOI:** 10.1080/17482631.2020.1756686

**Published:** 2020-04-28

**Authors:** Shih-Chun Lin, Mei-Chih Huang

**Affiliations:** a Department of Nursing, College of Medicine, National Cheng Kung University, Tainan, Taiwan; b National Tainan Junior College of Nursing, Tainan, Taiwan

**Keywords:** Spiritual practices, parents, end-of-life, decision making, brain stem dysfunction, qualitative

## Abstract

**Background:**

Adolescents with brain stem dysfunction may undergo many invasive treatments, and parents are often faced with making the decision to withdraw treatment. However, in the face of their child’s death, the spiritual practices of parents dealing with end-of-life decision-making remain under investigated.

**Purpose:**

This study explores the spiritual practices in parents making end-of-life decisions for adolescents on life support with brain stem dysfunction.

**Method:**

A descriptive phenomenological study was conducted through in-depth interviews with three parents of two adolescents in Taiwan. Data were analysed using Colaizzi’s seven-step protocol.

**Results:**

Three main themes emerged: (1) faith during decision-making, (2) struggles during decision-making, (3) transformation during decision-making. The findings indicate that “transforming the nature of hope” is the essence of the experience.

**Conclusion:**

Family-centred care, gaining insight into parental spiritual practices, and developing culturally-appropriate care are recommended.

## Background

Previous studies have described the support that religion and spirituality provide to parents when they are making difficult medical care decisions for their critically ill child (Allen, 2014; Ahmed et al., 2006; Chaplin et al., 2005; Koenig, 2002, 2009; Meyer et al., 2002; Michelson et al., 2009; Robinson et al., 2006; Superdock et al., 2018). Religion and spirituality comfort and strengthen parents (Seeman et al., 2003; Superdock et al., 2018), help them believe in miracles (Allen, 2014; Boss et al., 2008), and allow them to delay their acceptance of the harsh reality of their situation (Superdock et al., 2018), allowing them to be more optimistic (Nita, 2019) and less depressed (Madrigal et al., 2016). Yet, most of what is known about the support of religion and spirituality during end-of-life decision-making comes from studies performed in Western religious contexts (Bally et al., 2014; Bülow et al., 2012; Hexem et al., 2011; Kylmä & Juvakka, 2007; Superdock et al., 2018). How spiritual practices in East Asian religions influence parental end-of-life decision-making for their adolescent children has received far less attention.

Adolescents with brain stem dysfunction are usually in complex situations (e.g., multiple organ failure) and depend on life-sustaining treatments (e.g., artificial ventilation, vasopressors, blood products) to prolong their lives. Yang and Miller (2015) found that due to culture and religion, Asians are less likely to accept the concept of brain death. Occasionally, there may be conflicts regarding acting in the best interest of these adolescents (Royal College of Paediatrics and Child Health, 2004), where professionals consider further life-sustaining treatments to be untenable, yet, based on their religious beliefs, parents reject the advice of healthcare professionals and hold on to the possibility of a cure or choose to wait for a deity to cure their child (Liu et al., 2014; Nicholas et al., 2016).

Spiritual practice is defined in this study as an action or practice focusing on an internal and external sense of connection to something outside the self, such as a higher being, for the purpose of good outcomes and strength. These practices are diverse based on parents’ religion and culture and include prayers, rituals, practicing mindfulness, taking part in religious observances, or talking or confessing to one another in meaningful ways (Nita, 2019; Superdock et al., 2018). There are a total of 12,271 registered temples in Taiwan (Ministry of the Interior, 2019), and 38.5% of the Taiwanese population practice folk religions (Fu, 2018). Folk religions in Taiwan are in the category of polytheism, and are usually a mixture of Confucianism, Buddhism, Taoism, or other traditional Chinese religions. Studies have found that religious parents (Bülow et al., 2012; Phelps et al., 2009; Yun et al., 2011) or Asian parents (Ko et al., 2012; Phua et al., 2015) hold more favourable attitudes towards life-sustaining treatments. However, there have been no studies exploring parental end-of-life decision-making based on Taiwanese folk religions. Perspectives on end-of-life parental decisions related to the long-term survival of a child with sudden onset brain stem dysfunction are lacking. Improving our understanding of what struggles parents deal with as they choose to prioritize sustaining a child’s life, what their perceptions related to their spiritual practices are when facing end-of-life decisions, and how spirituality influences their decision-making will help providers provide guidance and support (Superdock et al., 2018). Further, it will prevent parental regret about the decisions they have made and improve end-of-life care (Phua et al., 2015). This study was an attempt to explore parental experiences in terms of spiritual practices when they face end-of-life decisions for adolescents with brain stem dysfunction on life support.

## Methods

The goal of this study is to explore what parents experienced and how it was experienced when they engaged spiritual practices when facing end-of-life decisions for their child in order to gain an understanding of the way they perceive and understand the end-of-life situation of their child and the meaning of their spiritual practices. A descriptive phenomenological method was used in this study. Parents were deemed eligible if (1) their child was on life support with brain stem dysfunction in a paediatric intensive care unit at a medical centre and (2) had participated in the child’s medical decision-making. A purposive sample of four parents of three adolescents met the inclusion criteria. The researcher approached the parents as a nurse and obtained their permission before engaging in the child’s end-of-life care. This process helped the researcher build a rapport with the parents.

Parents who met the inclusion criteria were approached by telephone after the child’s death. One parent refused to participate due to considering the interview to be too burdensome. Three parents of two adolescents with brain stem dysfunction based on a physician’s diagnosis were recruited.

Using an interview guide (Table I), data were collected by S.-C. Lin. through face-to-face, in-depth, individual interviews. All parents were interviewed within nine months after the child’s death. The interviews took place at the parents’ homes or restaurants where there was a private place to talk and lasted 60 to 120 minutes. The first author (S.-C. Lin) conducted the interviews, visited the participants and the places where they engaged spiritual practices, and analysed the data, with substantial contributions from the second author (M.-C. Huang). Both researchers had a paediatric nursing background and competency in end-of-life communication skills. The second author was experienced in conducting qualitative research. The parent interviews started with general questions, for example, by asking, “What is your understanding of your child’s illness and treatments? ” and “What did you find supported you to make difficult decisions?” and allowed the flow of conversation to be directed by the parents. When the parents mentioned involvement in or support from their religion or any spiritual practices, the researcher invited the parent to explain the detailed actions and processes involved in the spiritual practices and encouraged parental reflection on the motivations, experiences, beliefs, expectations, and challenges related to these spiritual practices when faced with an end-of-life decision for their child, for example, by asking, “How did you feel when you engage in spiritual practices in the situation you have just described?” “What did you expect from the spiritual practices?” and “Did you experience changes in your expectations for the child?” All interviews were audio-recorded and fully transcribed, including nonverbal language. The names assigned to each parent were pseudonyms. Data were managed using Atlas.ti software and analysed using Colaizzi’s (1978) seven-step protocol. First, the first author read the overall interview verbatim. Then, the researcher extracted significant statements from the verbatim transcription, as well as wrote the summary and reflection for each statement in order to reveal the researcher’s subjective views. Next, the researcher formulated meaning units from each statement. All the formulated meanings were organized into clusters of subthemes and themes and coded through repeated reading of the original verbatim transcript to ensure no statement was left out of the analysis. These subthemes and themes were integrated into an exhaustive description of the phenomenon of parental spiritual practices. Next, the researcher condensed and formulated the exhaustive description into a statement of the fundamental structure and discussed this with the second author to establish a consensus. The interview data were also triangulated with other data sources, including the researcher’s clinical logs, care reports, and process recordings during the last three months of patient care. Member-checking was employed by discussing the research findings, including the themes, subthemes, and descriptions, with each parent, and asking for feedback. This process allowed clarification, elaboration, and validation of the final explication of the findings by all of the parents in order to enhance the trustworthiness of the findings. In addition, peer-debriefing was employed by asking the members of the research team to review the research process, transcript, and the findings to enhance the quality of the data analysis. The peers in the research team included paediatric nursing scholars, qualitative research methodology specialists, a paediatric palliative care specialist, and paediatric nursing master’s students.

**Table I.** Interview guide.

Describe how you engage in spiritual practices. Describe your relationship to the Deity or the medium. Tell me about your feelings when engaging in spiritual practices. What problems did you face when making the most difficult decision? Describe how the Deity or the medium supported you? Tell me about your expectations of your spiritual practices. Did you experience changes in your expectations for the child? What does your child’s death mean to you?

## Ethical approval

Ethical approval was obtained from the National Cheng Kung University Hospital Institutional Review Board (A-ER-105-485). Written informed consent was obtained from all participants before the study.

## Findings

A total of three parents of two adolescent girls participated in the interviews. Two adolescents, Yu and Ping (psuedonyms), without a history of chronic illness, were transferred to the PICU due to clinical deterioration after Emergency Department admission to a medical centre. Both were diagnosed with brain stem dysfunction, one caused by severe brain oedema and the other caused by sepsis-associated multiple organ failure. All of the parents refused brain death examinations and requested that life-sustaining treatments be continued until cardiac death. The time interval from the point at which the child received life-sustaining treatment to death ranged from three to six months. The three parents were from southern Taiwan and ranged in age from 42 to 51 years old. Yu’s parents lived in a suburban area, and Ping’s mother lived in an urban area. The father worked full time, and the two mothers were housewives. All participants were polytheistic, worshipping deities recognized in the Taiwanese folk religion. Yu’s parents practiced in a local temple everyday, and Ping’s mother practiced at a home shrine almost everyday. All participants requested answers from a deity through medium conveyance or casting moon blocks (“bua buay”). The parents’ characteristics are described in Table II. Each participant was interviewed 2–3 times, with interviews ranging in length from 5–9 hours. Three themes emerging from the data in relation to spiritual practices when facing end-of-life decisions for a child were as follows: faith during decision-making, struggles during decision-making, and transformation during decision-making (Table III).

**Table II.** Characteristics of parents interviewed.

Characteristics	Adolescents of parents interviewed (n = 2)	Parents (n = 3)
Age in years, median (range)	15 (14–15)	47 (42–51)
Females, *n* (%)	2 (100)	2 (67)
Marital status, *n* (%)		
Married		2 (67)
Divorced		1 (33)
Religious affiliation, *n* (%)		
Folk beliefs in Taiwan		3 (100)
Employment status at death of the child, *n* (%)		
Off work		2 (67)
Number of children per families, *n* (%)		
1	1 (50)	1 (33)
4	1 (50)	2 (67)
Palliative shared care, *Yes* (%)	1 (50)	
DNR order signed within 24 hours of death, *n* (%)	2 (100)	
Place of death, *n* (%)		
Intensive care unit	2 (100)	
Time between receiving life-sustaining treatment and death in months median (range)	4.5 (3–6)	
Duration of interview in hour; median (range)		6.3 (5–9)

**Table III.** Themes and subthemes of spiritual practices identified by parents facing end-of-life decisions for their child on life support with brain stem dysfunction.

Theme	Subtheme	Meaning unit
Faith during decision-making	1. Not giving up on life	1. Encouraging the child not to give up living. 2. Trying every possible measure to save the child. 3. Fearing a loss of opportunities to save the child.
	2. Expecting a miracle	4. Waiting for positive outcomes or miracles to occur. 5. Witnessing a miraculous recovery.
Struggles during decision-making	3. Being forced to make decisions	6. Having no choice other than invasive treatments to prevent the child’s death. 7. Complying with medical recommendations and hospital restrictions.
	4. Sense of the incomprehensible	8. Lacking an understanding of the illness. 9. Not understanding the deity’s purpose.
	5. Hatred, regret, and guilt	10. Unforgiving to those felt to be responsible for the child’s illness. 11. Deeply regretting what could have been prevented. 12. Feeling guilty due to feeling responsible for the death.
Transformation during decision-making	6. Turning to the child’s intentions	13. Considering the child’s suffering. 14. Considering the child’s characteristics and preferences.
	7. Transformation to a harmonious end of life	15. Changing expectations of the child’s recovery. 16. Accepting the reality of the child’s death. 17. Seeking a harmonious end to the child’s life.

## Theme 1: Faith during decision-making

The parents had faith about their decision-making when they engaged in spiritual practices. They all believed that folk deities have the power to heal their child and therefore decided to continue life-sustaining treatments even though from the health care professionals’ perspectives, the actual burdens on their child were felt to outweigh the benefits.

### Not giving up on life

The parents indicated that due to their understanding of the severity of the illness, they tried everything they could and prolonged the treatment to wait for a folk deity to heal their child. The parents searched for folk therapies, utilized acupoint magnetic therapy and dietary supplements, engaged in ancestor worship, fed the child a mixture of water and burnt charm paper, or expected “the heavenly military” to protect the child, as suggested by other relatives, friends, or mediums. These parents believed that combining the use of spiritual practices with conventional medicine and intensive care would be the best way to treat the child.

‘Sometimes I pray and sit in meditation at the medium’s place. Meditation makes me calmer … … like pouring chi down over the top of my head, so I could transfer chi to her [Ping] … … in the past half year, we did what we could.’ (Ping’s mother)

‘Little by little, I kept fighting for time, cause I knew that if I didn’t hang onto the opportunity, I would miss the chance again … ….I would never give up on her until the white cloth covered her body [death]!’ (Yu’s father)

Yu’s father said that although the healthcare providers showed him the flat electroencephalogram, trying to convince him of the poor prognosis, he believed the “deity’s eyes could see better than all medical devices.” When asking the parents perspectives about withdrawing or withholding the child’s life-sustaining treatments, all the parents objected to this option and mentioned about their philosophy that “life should be cherished,” “though appearing threatening, all crises will be survived one after the other.” For some parents, the concepts of “hospice” and “do-not-resuscitate” indicated they had given up on their child. Suggestions along these lines made the parents feel abandoned and caused them to doubt whether the healthcare providers truly cared about their child, which resulted in a lack of trust in the parent-healthcare provider relationship.

### Expecting a miracle

While to healthcare professionals, brain stem dysfunction with multiple organ failure ensures that death is imminent. From the parents’ perspective, since the child had survived after cardiopulmonary resuscitation and narrowly escaped death, the parents were hopeful that the child was blessed, and this made them feel that a miracle could occur. Folk deities provided parents through their spiritual practices with more positive information in a “constructive” manner, such as “promise to send the heavenly military to protect the child” or “prescribe medicine from the Deity.”

‘What a miracle! Usually people can’t be resuscitated after an hour. However, her heartbeat returned after an hour … … I would like to give her a chance, maybe there will be a miracle.’ (Ping’s mother)

‘The Deity said [as conveyed by the medium] she had personally gone into her body to help her. Sometimes we saw movement [the child’s] cheek. It seems there were miracles (looking satisfied).’ (Yu’s mother)

Sometimes parents felt burdened and annoyed when the healthcare providers kept informing them of the poor likelihood of survival. Therefore, they turned to a deity for more optimistic information, hopeful communication, and self-affirmation. One father described himself as “feeling energized” and ‘full of hope and expectations” even though he was in a state approaching physical exhaustion. The parents kept searching for hopeful clues, including remembering that they had felt that the child was special since she was born and searching for positive stories in the news. They also observed the child’s vital signs and symptoms or searched for hopeful messages from the healthcare providers, such as “have never seen one like her who has lived so long” to confirm their hope.

Some parents didn’t want their healthcare providers to give them bad news in front of the child with brain stem dysfunction due to believing that it would bring despair and threaten the child’s hope. They believed that conveying a positive attitude to the child suggesting that “humans should fight against all life threats and challenges,” would lead to a greater chance that their child would survive.

## Theme 2: Struggles during decision-making

The parents struggled with making difficult decisions through engaging in spiritual practices. They were faced with the dilemma of whether to follow the directions of healthcare professionals or folk deities.

### Being forced to make decisions

All parents described the experience of having no choice but to make hard decisions. A feeling of pressure was described by some parents who felt they had to comply with medical recommendations or involuntary treatments for their child despite the fact that the providers may have asked the parents their preferences in this regard.

‘We were really afraid that they would not ponder over her [their child’s] treatments after we made a “do not resuscitate” decision for her … … However, to comply with the rules of the hospital, we had to make that decision.’ (Yu’s mother)

Conflicts and frustration occurred when the strong recommendations from the professionals were different from or opposite that of the will of the folk deities.

‘They [the professionals] kept saying that if our daughter did not have tracheal intubation, she would be in danger … … but our Deity opposed! … .the hospital kept putting pressure on us, so we had no choice (but to agree with the insertion of a tracheal tube).’ (Yu’s mother)

The parents had to make some decisions because other treatment options could potentially hasten the child’s death.

‘How could we choose to stop hemodialysis? She [the child] would definitely die if she did not receive hemodialysis!’ (Yu’s father)

### Sense of the incomprehensible

Spiritual practices offer parents an alternative to biomedical explanations of illness by describing the period when the child was being resuscitated as, “two of the three souls temporarily leave the body,” and stating that their child was “in a deep trance” or “locked-in her body.” Disconnected communication between parents and the healthcare providers occurred when the medium and the healthcare providers had different explanations for the severity and extent of the illness, prognosis and treatment. Ping’s mother stated that even after attending a family meeting, she still felt the burden of piecing together all the information that she had been given.

‘I’ve tried many ways to understand why she was brain dead … … I think the doctor should tell me that because we are just normal people and don’t understand.’ (Ping’s mother)

Some parents asked “why the child could not escape the fate of death,” expecting that there would be a reason, e.g., the deity wanted to give the parents a lesson; if they got through it, their child might survive. Due to acknowledging their uncertainty and fear of making wrong decisions, the parents expressed concern about “whether the decision was truly good for her.” Therefore, they sought solace and direction from the folk deities to reassure themselves that they had made the right decision and that they had sustained a supportive relationship until the child’s death and beyond.

‘We consulted with the Deity before making decisions. Whenever the doctors told us something, we would think again and again about how to deal with it … … We always hesitated over the decisions we made.’ (Yu’s mother)

### Hatred, regret, and guilt

The parents experienced hatred, regret, and guilt when they practiced folk religions, and their expectations were not realized. They experienced hatred towards the people and events that were related to the child’s death, including the schoolteachers, physicians, the Deity, and family. Ping’s mother expressed resentment related to what was perceived to be medical negligence and planned to sue the hospital and physician for the child’s injury. Yu’s parents had a hatred of the Deity for not bringing peace and health to the child.

*‘*We wrote a whole paper of questions! … … I can’t say I’m not angry (toward the Deity), but I want to ask why? I didn’t pray for wealth and prosperity, I just wanted to keep my child safe and sound.’ (Yu’s father)

All of the parents showed regret and guilt associated with the perception that they and their families may have contributed to the child’s injury and death. They expressed regret related to spending too much time at work, not detecting the child’s discomfort earlier, or not pushing physicians hard enough to agree to the hospital transfer, which may have fuelled their determination to be more cautious in the future. These states of guilt were reflected in their thinking about stopping prolonging the child’s life and letting the child die or a belief that they had done something bad in the past that might have resulted in their child being cursed.

‘I feel guilty about her. It fell to her lot to be cursed for the family’s bad luck.’ (Yu’s father)

## Theme 3: Transformation during decision-making

During the process of praying, asking questions, meditating, and reflecting, spiritual practices were necessary for parents to alter the way they perceived and construed their child’s death. The child’s intentions were recalled, and this encouraged the parents to feel that they were making the right decisions.

### Turning to the child’s intentions

Since the child’s feelings related to whether they had the desire to live or hasten death were critical for them to make decisions, the parents chose to perceive their child’s intentions through dreaming, observing, recalling past experiences, finding any trace of the child’s preferences, or asking the folk deities for answers. Ping’s mother strongly believed that her daughter had the will to survive when she decided to continue Ping’s life-sustaining treatments. Yu’s parents generally encouraged Yu to maintain hope, yet when observing her suffering over time, they felt that “even a normal person would probably commit suicide,” which made them wonder whether Yu still had the desire for life to continue. The interpretation of the child’s intentions gave parents explanations and answers related to their child’s “choice” to die.

‘The Deity said [as conveyed by the medium] that she wanted to leave [die] after a month and a half … … we think this is possible. She had once told her sister when she was watching the news about an earthquake, that she would rather die than live like those who had lost their limbs.’ (Yu’s mother)

‘Her [Ping’s] uncle said that he had dreamed of his deceased brother coming back, who told him that she [Ping] was going to wait in line to reincarnate … … When I told her to let go and practice religion in heaven, it was true that her blood pressure started to drop.’ (Ping’s mother)

### Transformation to a harmonious end of life

Although the parents reported that their goals of care for the child were always “to cure” the illness, their expectations of the child’s recovery shifted over the course of time depending on the illness and situation. Yu’s parents reported their hope changed from “to cure her completely … … return her health, and let her walk to school” to “if she could not wake up … … at least she could survive.”

The parents described that taking every measure to save the child and finding their efforts to be all in vain, made them feel “there is no other way,” and “there are no more miracles.” Witnessing the child’s pre-dying signs was a major trigger for them to start to believe that this was their last chance to save their child and that there was a high likelihood that their child would die. The signs of death parents observed included coolness, darker skin and lip colour, and changes in their outer appearance. This, combined with the poor physiological data the healthcare providers had reported, caused the parents to feel overwhelmed, as if they had emotionally “fallen down the stairs” and “had been sentenced to death,*”* which led to their understanding that death was imminent.

‘Like suddenly … … like I was falling down … … she had already passed away, what else do I ask for?’ (Yu’s father)

All of the parents in this study relied on a deity to give the child a harmonious end of life by worshipping folk deities in a temple or at home. Their belief that their child had come to a harmonious end was reflected in the ways that they described their child using phrases such as “peace,” “destined blessing,” “a better place,” “become immortal,” “never reincarnate.” Parents were grateful to the folk deities for showing mercy to their dying child, and felt assured that the child would be kept save and have a blessed afterlife. The spiritual practices provided parents with “acceptance” and helped them “feel a connection with the child” that led to comments such as, “We know where to find her.”

‘The Deity left us speechless when he told us two things [as conveyed by the medium]. First, he felt sorry for us … … Second, he had already taken her [Yu] at his side … … The Deity blessed my child … … there will be no more pain … It’s good to know that my child is staying with the Deity now.’ (Yu’s father)

‘He [the medium] said that if I live well, I will be able to meet her [Ping] in the future.’ (Ping’s mother)

## Discussion

Three main themes emerged from the data related to the parental experience with spiritual practices as they faced end-of-life decision-making, including: (1) faith during decision-making, (2) struggles during decision-making, and (3) transformation during decision-making. Their spiritual practices were described as religious guidance from folk deities, where “transforming the nature of hope” was the essence of the experience for parents believing in the Taiwanese folk religion as they made end-of-life decisions for their adolescent child on life support with brain stem dysfunction. There are some similarities between the themes found in this study and those discussed in previous studies, which were focused on spirituality and religion in Western parents, including “accepting reality” and “restructuring of feelings of hope,” expressed by parental caregivers who had children in treatment for cancer (Bally et al., 2014). There were “hope and faith,” “the meaning of suffering,” and “the meaning of death” themes in Christian parents in the USA who made decisions for their infants with complex life-threatening conditions (Superdock et al., 2018).

There are differences in the experiences and needs between parents making end-of-life decisions for their adolescent child. First, while parents of a child receiving cancer treatment experienced moving from “preparing for the worst” to “hoping for the best” (Bally et al., 2014), the parents in this study avoided discussing future plans for care, such as withdrawing or withholding life sustaining treatment or for their child not to be resuscitated, when their child’s condition deteriorated and reported that this would threaten their hope for the child’s recovery. They reported feeling depressed and hopeless about receiving daily “bad news” and feeling overwhelmed with the stressful information overload related to an uncertain prognosis. In addition, they felt that they were pressured into making decisions against their will.

Second, the concerns of parents and the factors that influence decision-making were different between parents. In previous studies, parental recognition of their child’s suffering and poor quality of life weighed heavily in their choice to withdraw life-sustaining treatments and to allow the child to die naturally due to the progress of their underlying disease (Allen, 2014; Michelson et al., 2009; Xafis et al., 2015). However, in this study, as death could be predicted following withdrawing or withholding life-sustaining treatments, this would not have been considered by the parents. They preferred to maintain the child’s life regardless of the quality of life and the minimal chance of recovery. The benefit of continuing life and waiting for a medical cure or a miracle from a deity to cure the child outweighed the burden of treatment in the case of these parents.

Third, reliance on the knowledge and opinions of healthcare professionals in decision-making was different between parents. Parental hope for a child with cancer is typically built largely on their faith in the oncologist (Salmon et al., 2012). The parents in this study not only asked the healthcare professionals to explain all the end-of-life decisions, but also sought the advice of folk deities. Yu’s parents reported that they trusted a deity more than the healthcare providers in terms of decision-making. Instead of emphasizing the limitations of medical technology, their faith emphasized the ability of a deity to cure and protect the child, which was congruent with the parents’ sense of hope and their philosophy of life.

Forth, the findings from this study indicated that end-of-life decisions are not only made between parents and healthcare professionals, but that mediums also influence these decisions. Struggles occurred when the parents felt the decisions were not always according to their will, or when the healthcare professionals had different views from that of the deity they were consulting. Many healthcare providers may feel helpless when parents rely heavily on folk deities to make decisions, especially when such decisions are in conflict with medical evidence.

Finally, this study adds an understanding of the role of spiritual practices for parents while facing end-of-life decision-making. Previous studies regarding spiritual care for parents have highlighted their need to maintain hope as they accept their child’s prognosis (Allen, 2014; Cutcliffe & Herth, 2002; Fitzgerald Miller, 2007; Stevenson et al., 2013; Superdock et al., 2018; Weaver et al., 2016). Parents’ faith in the control of a deity not only gave them strength, but also led to a justification to delay acceptance of harsh realities and to continue holding on while hoping for a good outcome (Superdock et al., 2018). Folk religion in this study offered an alternative to biomedical explanations and scientific treatments of illness, and helped convey the thoughts, feelings, and intentions of the unconscious child to their parents. Although hope related to the child’s physical wellness seemed unrealistic towards end-of-life, the process inspired hope and involved unconditional acceptance, tolerance, understanding, and caring (Cutcliffe & Herth, 2002; Fitzgerald Miller, 2007) and as such, is relevant to this study and further work in relation to the role of folk deities in decision-making.

Spiritual practices played a pivotal role in promoting faith by creating the means and beliefs by which to have such faith, which was central and essential for these parents to deal with their child’s illness and to continue their lives with value and meaning. Healthcare providers should be aware of parental stressors during decision-making, identifying parental needs as both care providers and care recipients, in order to minimize their stress, preserve their well-being, and provide family-centred care (Richards et al., 2017; Shudy et al., 2006).

### The strengths and limitations of the study

This is the first study to explore the parental experience related to spiritual practices when they are facing end-of-life decisions for a child on life support with brain stem dysfunction. Given that the end-of-life decision is culturally sensitive, the methodology was strengthened by interviewing parents with whom the researcher had direct involvement in the care of their child for at least three months. This process was helpful for the researcher to enhance rapport and built trust with the parents. This also helped the researcher to understand the clinical situations the parents were facing as well as the interactions between the parents, healthcare providers, and mediums. In addition, by personally interviewing the parents in their homes and community, the researcher was better able to understand their family’s culture.

There are several limitations to this study. First, the study is based on the experiences of three parents of adolescents on life support with brain stem dysfunction. The parents had been informed by the physicians that the child might be in a brain dead state even without performing brain death examinations; therefore, the findings might not represent the experience of other parents of adolescents with complex chronic or life-threatening conditions. Second, the parents in this study strongly favoured the continuation of life-sustaining treatments until the child’s death and believed in their Taiwanese folk religion. These findings might not represent the experience of parents prioritizing comfort even at the expense of sustaining life. In addition, the experiences described here might not represent parents who are not folk religionists or whose spiritual beliefs are not a guiding factor in their decision-making.

### Implications for practice with families

Regarding parental end-of-life decision-making, this study widens current knowledge beyond that of dominant Western worldviews. Spiritual practices can be viewed as the expression of a family’s culture and belief system that guides parental decisions and actions and therefore has a strong connection with healthcare and their overall wellbeing. The results of this study can be applied to explore experiences of end-of-life decision-making based on different cultural backgrounds. Further investigation of care needs among cultural minorities facing end-of-life decisions is needed.

Parents need to maintain and transform the nature of hope despite underlying differences in culture and religion. Spiritual practices not only create an opportunity for faith during decision-making, but also give parents a way to do everything possible in terms of both physical and religious care for their child. There are no guidelines suggesting how to handle circumstances in which parents heavily rely on spiritual practices for making end-of-life decisions. A panel of paediatric critical care specialists, palliative care specialists, ethicists, and spirit mediums in folk religions is recommended to develop a model of care for managing these socially and ethically complex situations in order to avoid conflict and struggle among parents and healthcare providers.

## Conclusion

The results indicated that parents believing in a Taiwanese folk religion rely heavily on spiritual practices as they make end-of-life decisions for their child with brain stem dysfunction. In the face of an increasing multi-cultural society, spiritual practices for parents are becoming increasingly diverse, and end-of-life decision-making for a child should be underpinned by an understanding of the diversity of the parents’ own moral values and beliefs. Understanding the meaning behind parental spiritual practices can help healthcare providers understand what’s important to them. Further work to develop and evaluate a culturally-appropriate model of care in Taiwan that involves the opinion of the spirit medium in folk religions for best practice family-centred care is recommended.
